# EMD-Based Symbolic Dynamic Analysis for the Recognition of Human and Nonhuman Pyroelectric Infrared Signals

**DOI:** 10.3390/s16010126

**Published:** 2016-01-20

**Authors:** Jiaduo Zhao, Weiguo Gong, Yuzhen Tang, Weihong Li

**Affiliations:** 1 Key Lab of Optoelectronic Technology and Systems, Chongqing University, 174 Shazheng Street, Chongqing 400044, China; jdzhao@cqu.edu.cn (J.Z.); weihongli@cqu.edu.cn (W.L.); 2 Technology Center of Sichuan Changhong Electric Co. Ltd, 199 Tianfu Road, Chengdu 610000, China; 2008tangyzchongda@gmail.com

**Keywords:** empirical mode decomposition, symbolic dynamics, pyroelectric infrared signals, feature extraction, pattern classification

## Abstract

In this paper, we propose an effective human and nonhuman pyroelectric infrared (PIR) signal recognition method to reduce PIR detector false alarms. First, using the mathematical model of the PIR detector, we analyze the physical characteristics of the human and nonhuman PIR signals; second, based on the analysis results, we propose an empirical mode decomposition (EMD)-based symbolic dynamic analysis method for the recognition of human and nonhuman PIR signals. In the proposed method, first, we extract the detailed features of a PIR signal into five symbol sequences using an EMD-based symbolization method, then, we generate five feature descriptors for each PIR signal through constructing five probabilistic finite state automata with the symbol sequences. Finally, we use a weighted voting classification strategy to classify the PIR signals with their feature descriptors. Comparative experiments show that the proposed method can effectively classify the human and nonhuman PIR signals and reduce PIR detector’s false alarms.

## 1. Introduction

The pyroelectric infrared (PIR) detector has been widely used in many industrial and security applications. It has many advantages, such as low cost, low power consumption and no invasion of privacy, which endow it with strong market competitiveness [1]. However, in the field of public security, due to its restrained target discriminating ability and vulnerability to environmental noise, its performance is often limited by high false alarm rates and classification errors [2]. Therefore, exploring effective feature extraction and classification methods for recognizing the human and nonhuman PIR signals has become a meaningful and interesting task.

In recent years, some researchers have used PIR sensors or sensor networks for target tracking [3], fire detection [4] and human identification [5], *etc.*, but only a few studies have attempted to explore an effective human and nonhuman recognition method with a single PIR sensor [6,7,8]. In [6], Wang *et al.* employed wavelet packet entropy (WPE) to capture the transient features of human and nonhuman PIR signals and declared that their method could recognize the adults, dogs and bulbs. Considering that auto-regressive (AR) spectrum analysis can obtain high frequency resolution, Gong *et al.* [7] used AR-based spectral estimation to extract the frequency features of the human and nonhuman PIR signals for reducing PIR detector false alarms. Besides, Xin *et al.* [8] adopted symbolic dynamic filtering (SDF) as the feature extraction method for target detection and classification using seismic and PIR sensors and obtained satisfactory results.

The methods mentioned above have indeed improved PIR detector performance, but they still have some drawbacks. Although the WPE-based feature extraction method can effectively capture the transient features of the original PIR signal, to achieve a high classification accuracy, the wavelet bases must be carefully selected. The AR model is a suitable tool for the analysis of the stationary signals, but PIR signals are sometimes non-stationary. SDF can effectively identify the statistical patterns of the symbol sequences generated from the time series data [9], however, the symbolization can be viewed as a coarse-graining of the original PIR signal, which may lose the detailed feature information contained in the original PIR signal [10]. Besides, these studies only provide signal processing methods for human and nonhuman recognition, but they never analyze the inherent differences between human and nonhuman PIR signals or use these differences to explore a more effective recognition method.

To address the problems mentioned above, first, we build a mathematical model of the PIR detector for analyzing the discriminable features of the human and nonhuman PIR signals and finding their inherent differences. Then, according to the analysis results, we propose an empirical mode decomposition (EMD)-based symbolic dynamic analysis method for effectively extracting the discriminable features of human and nonhuman PIR signals. In the proposed method, firstly, we propose an EMD-based symbolization method to rudimentarily extract the detailed features of the original PIR signals into five symbol sequences. Secondly, through constructing multiple probabilistic finite state automata (PFSA) with the symbol sequences, we generate five feature descriptors for each PIR signal. Finally, using a weighted voting classification strategy, we integrate the feature information contained in the feature descriptors and classy the original PIR signal.

The rest of the paper is organized as follows: Section 2 discusses the physical characteristics of the human and nonhuman PIR signals. Section 3 describes the proposed method. Section 4 evaluates the performance of the proposed method in two different applications. Section 5 gives the conclusions of this paper.

## 2. Physical Characteristics of Human and Nonhuman PIR Signals

**Figure 1:**
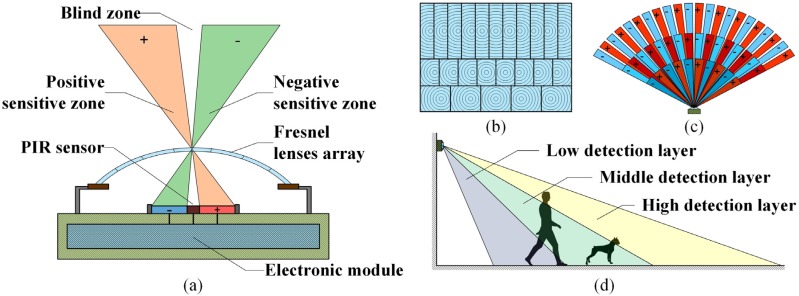
(**a**) Structure of a common PIR detector; (**b**) layout of the Fresnel lenses array; (**c**) top view of the distribution of the SZs and NZs in each detection layer, where the sectors labeled by “+” indicate the PSZs, those labeled by “−“ indicate the NSZs and the gaps between the PSZs and NSZs indicate the BZs; (**d**) lateral view of the distribution of the three detector layers.

To find an effective human and nonhuman PIR signal recognition method, firstly, we analyze theoretically their discriminable features using a mathematical model of a PIR detector. As shown in Figure 1a, a typical PIR detector usually consists of a Fresnel lenses array, a PIR sensor and an electronic module.

The Fresnel lenses array partitions the detector’s field of view (FOV) into a series of alternately distributed “sensitive zones” (SZs) and “blind zones” (BZs). Owing to the dual structure of the PIR sensor, the SZs are divided into positive SZs (PSZ) and negative SZs (NSZ) [11]. If a thermal source goes from a PSZ into a NSZ, the polarity of sensor’s output will be reversed. Because of the three-row layout of the Fresnel lenses array, as shown Figure 1b, the FOV of the PIR detector is accordingly divided into three detection layers, as shown in Figures 1c,d, each detection layer contains different number of SZs (or NZs). Because of the different heights and body shapes, the human and nonhuman targets would intersect with different detection layers when they cross the detector’s FOV. To find the relationship between the intersection pattern of the target and the characters of its PIR signals, we build a mathematical model of the PIR detector. When a PIR sensor is heated by a thermal source, it would generate a current signal that satisfies Equation (1) [12,13]:

(1) $$I\left(t\right)=pA_{s}\frac{d}{dt}T\left(t\right)=\frac{pA_{s}\alpha }{C_{p}}\exp\left(-\frac{G_{Th}t}{C_{p}}\right)u\left(t\right)*\frac{d}{dt}\Phi \left(t\right)$$

where *t* indicates the time instance, *p*, *C_p_* and α indicate the pyroelectric coefficient, heat capacity and absorptivity of the sensitive element respectively, *T*(*t*), *A_s_* and *G_Th_* separately indicate the temperature, surface area and thermal conductance of the PIR sensor, *u(t)* is the unite step function, ∗ is the convolution operator, and **Φ**(*t*) is the thermal power received by the PIR sensor. According to Liu’s study [13], based on the Stefan-Boltzman law, **Φ**(*t*) can be simplified as Equation (2):

(2) $$\Phi \left(t\right)=\frac{k_{B}\varepsilon_{h}\left(T_{a}^{4}-T_{s}^{4}\right)}{d_{0}^{2}}A(t)+N(t)$$

where *d_0_* indicates the distance between the thermal source and the PIR sensor, *k_B_* indicates the emissivity of the thermal source, *ε_h_* is the Stefan-Boltzmann constant, *T_a_* and *T_s_* indicate the temperature of the ambient and the the thermal source respectively, *N*(*t*) is the thermal noise and *A*(*t*) is the surface area of the thermal sources that can be observed by the PIR sensor. In the PIR detector, because the infrared radiation coming from the PSZs would be offset by those coming from the NSZs [11], A(*t*) actually equals to the difference between the target’s surface area that exposes in the PSZs and that exposes in the NSZs, *i.e.*,:

(3) $$A\left(t\right)=\sum_{i=1}^{N}A_{i}^{+}\left(t\right)-\sum_{i=1}^{N}A_{i}^{-}\left(t\right)$$

where $A_{i}^{+}\left(t\right)$ is the targets’ surface area that exposes in the *i*th PSZ, $A_{i}^{-}\left(t\right)$ indicates the surface area that exposes in the *i*th NSZ, *N* is the number of the PSZs (or NSZs) in the entire FOV. Therefore, we can term *A*(*t*) as “net effective area” (NEA) and term its changing curve as the “NEA curve”.

**Figure 2:**
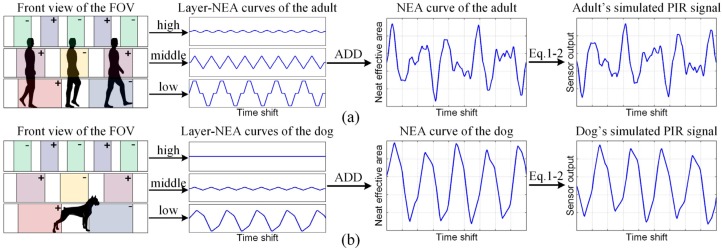
Simulation of the adult’s (**a**) and the dog’s (**b**) PIR signals, where the squares with the symbol “+” in the front view of FOV indicate the PSZs and those with the symbol “−“ indicate the NSZs, the blank between PSZ and NSZ indicates the PSZs.

Using the mathematical model elaborated above, we can simulate the generation process of the human and nonhuman PIR signals (taking an adult and a dog for instance) for analyzing their inherent differences. As shown in Figure 2, because the detector’s FOV is divided into three detection layers by the Fresnel lenses array, according to the targets’ body shapes and the width of SZs and BZs [7], we first calculate the “layer-NEA curves” of each target, *i.e.*, the changing curves of the surface area that exposes in each detective layer, rather than the entire FOV. We can see from Figure 2 that, even in the same detection layer, the adult’s and dog’s layer-NEA curves still have different patterns, this is because the adult and dog have different body widths. Through adding the three layer-NEA curves together, we can obtain the targets’ NEA curves in the entire FOV. We can see from Figure 2 that because the adult is much higher than the dog, he can intersect with more detection layers than a dog. Therefore, after adding the three layer-NEA curves together, the adult would generate a relatively complex NEA curve with many riding wives and small fluctuations, whereas the dog will generate a smoother one. Through substituting the NEA curves (*i.e.*, *A*(*t*)) and another sensor’s parameters into Equations (1) and (2), we can obtain the simulated PIR signals.

From Figure 2, we can see that the simulated PIR signals have the same characteristics with the corresponding NEA curves, which means that the adult’s PIR signals would have more riding waves and small fluctuations, whereas the dog’s one is much smoother. The analytical result indicates that the discriminable features of the human and nonhuman targets (*i.e.*, the body shape and height) are represented by the details of their PIR signals.

**Figure 3:**
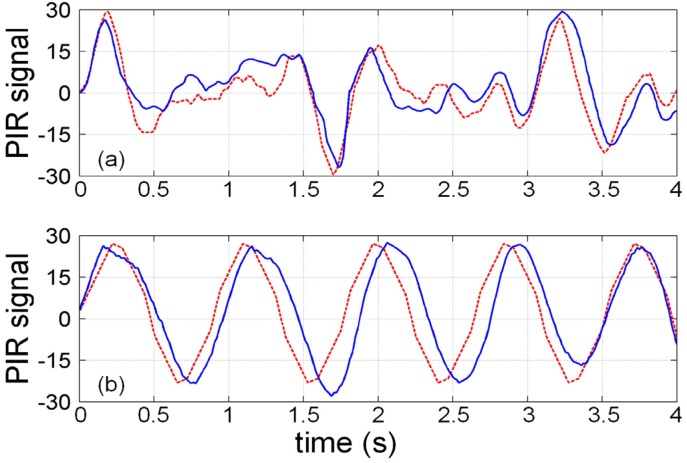
Real (blue-solid) and simulated (red-dashed) PIR signals of adult (**a**) and dog (**b**).

To verify the credibility of the analytical results, we collected 100 adults’ and dogs’ PIR signals under the same conditions as the simulation. As shown in Figure 3, we compared the collected PIR signals with the simulated results and calculate the average correlation coefficients between them. The average correlation coefficients between the simulated and collected PIR signals of the adults and dogs are 0.802 and 0.819, respectively, which proves the credibility of the analytical results.

## 3. EMD-Based Symbolic Dynamic Analysis

**Figure 4:**
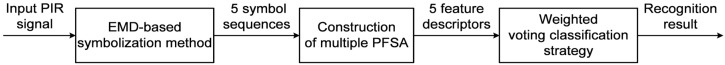
Constitution of the EMD-based symbolic time series analysis.

According to the analytical result mentioned above, we know that the key issue for the recognition of the human and nonhuman PIR signals is to effectively extract their discriminable features from their signal details. To do this, we propose an EMD-based symbolic dynamic analysis method. As shown in Figure 4, the proposed method mainly consists of three steps:

(1) Use an EMD-based symbolization method to generate five symbol sequences with the feature information of the original PIR signals.
(2) Construct multiple probabilistic finite state automata (PFSA) with the generated symbol sequences for generating five feature descriptors for each PIR signal.
(3) Use a weighted voting classification strategy to fuse the feature information contained in the feature descriptors and classify the original PIR signal.

### 3.1. EMD-Based Symbolization

Empirical mode decomposition is a novel nonlinear and non-stationary signal processing method [14,15] that has been widely used in many applications, such as fault diagnosis [16] and biomedical signal analysis [17]. It is built on the assumption that each complex signal is composed of a number of simple oscillation modes. According to the time scales of these oscillation modes, EMD can separate them into a number of simple fluctuations named intrinsic mode functions (IMF). The relationship between a complex signal *X*(*t*) and its IMF components can be described by Equation (4):

(4) $$X\left(t\right)=\sum_{i=1}^{N}C_{i}\left(t\right)+r_{N}\left(t\right)$$

where *N* is the number of total IMF components, *C_i_*(*t*) and $r_{N}\left(t\right)$ indicate the *i*th IMF component and the residue respectively.

Taking the advantage of EMD, we can isolate the details of the PIR signal from the original signal and decompose them into specific IMF components because their time scales are relatively smaller than another components. Then, through analyzing these IMF components, we can specially extract the discriminable features of the human and nonhuman PIR signals carried by the signal details. Following this line of thought, we present an EMD-based symbolization method to extract the detailed features of the original PIR signal and generate five symbol sequences for further analysis.

**Figure 5:**
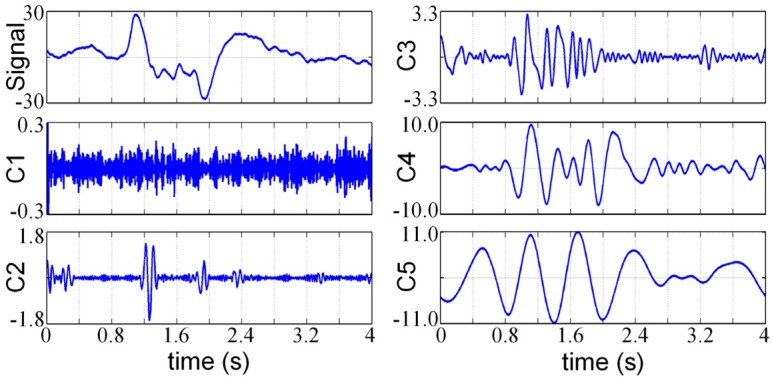
IMF components of an adult's PIR signal, where ***C****_i_* is the *i*th IMF component.

The first step of the proposed method is using the EMD to decompose the original PIR signal into a number of IMF components. Because the details of the PIR signals usually have small time scales and the EMD always arranges the IMF components in an ascending order of their time scales [14], thus the details of the original PIR signal would be primarily extracted into the first few IMF components. For this reason, we can just use the first five IMF components for further feature extraction. Figure 5 shows the decomposition results of an adult’s PIR signals, we can see that the details of the PIR signal have been successively decomposed into different IMF components and arranged in an ascending order of their time scales.

**Figure 6:**
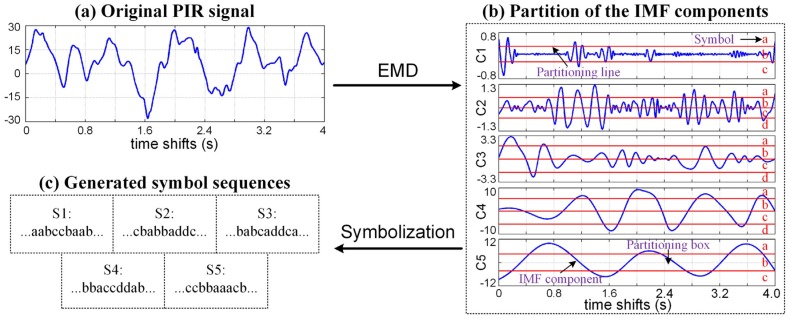
Illustration of the EMD-based symbolization method, where *C_i_* indicates the *i*th IMF component and *S_i_* indicates the symbol sequence generated from *C_i_*.

To extract the feature information contained in the generated IMF components, as shown in Figure 6, the second step of the proposed symbolization method is partitioning the data space of the IMF components for generating five symbol sequences. In the partitioning procedure, first, the data space of the *i*th IMF component ***C_i_*** is partitioned into a number of mutually exclusive and exhaustive regions using the maximum entropy partition method [18], which ensures that the information-rich regions of the IMF component can be partitioned finer and those with sparse information are partitioned coarser. Then each region is labeled by a unique symbol picked from an alphabet $\;\Sigma_{i}$. The choice of the alphabet size $\;\left|\Sigma_{i}\right|$ depends on the specific data set and experiments [19], so the alphabets adopted by different IMF components may have different sizes. For example, in illustration shown in Figure 6b, the alphabet used for the partition of ***C_1_*** is $\;\Sigma_{1}=\{a,b,c\}$ with the size of 3, whereas the alphabet used for partitioning ***C_2_*** is $\;\Sigma_{2}=\{a,b,c,d\}$ with the size of 4. At last, each data point in ***C_i_*** is assigned to a particular symbol according to the region where the data point falls, in this way, we can generate a symbol sequence ***S_i_*** from the *i*th IMF component. Through repeating this partitioning procedure on each generated IMF component, we can obtain five symbol sequences as shown in Figure 6c.

It has been indicated in [20] that the essential robust features of the original signal can be extracted into a symbol sequence through an appropriate partition, but these features still cannot be directly used for the classification. Therefore, further analysis is still needed to generate appropriate feature descriptors for the original PIR signal.

### 3.2. Construction of Multiple PFSA

To extract the features contained in a symbolic sequence, Ray [20] constructed a probabilistic finite state automat from a symbol sequence with an assumption that the symbol sequence satisfies the *D*th order Markov machine, and then he calculated the normalized left eigenvectors of the state transition matrix of the constructed PFSA and used them as the signal’s feature descriptor. The feature descriptor generated by Ray’s method can effectively represent the patterns represented by the symbol sequence [21], so we adopt Ray’s method for generating five feature descriptors from the multiple symbol sequences generated by the EMD-based symbolization method.

**Figure 7:**
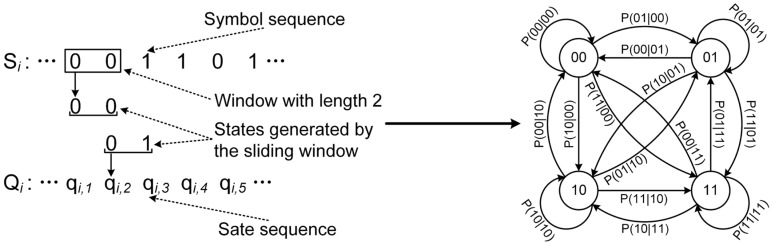
An example of constructing a PFSA from a symbol sequence, the left-hand of the figure indicates the generation of state sequence and the right hand indicates the constructed PFSA, in which the circle indicates the state of the PFSA and the arrow indicates the transition between the states.

As shown in Figure 7, to construct a PFSA from the symbol sequence ***S_i_***, we first put a sliding window with the length of *D* on the symbol sequence and treat the *D* symbols fallen in the window as a state of the PFSA. Through sliding the window along ***S_i_***, we can convert ***S_i_*** to a state sequence (termed as *Q_i_* = *q_i,_*_1_, *q_i,_*_2_,…).

Then we use the generated state sequence to estimate the probability of the transition between two states according to Equation (5):

(5) $$P({ο}_{i,k}|{ο}_{i,l})=\frac{P({ο}_{i,k}{ο}_{i,l})}{P({ο}_{i,l})}\;\approx \frac{N({ο}_{i,l},{ο}_{i,k})}{\sum_{j=1}^{\left|\Psi_{i}\right|}N({ο}_{i,l},{ο}_{i,j})}$$

where ${ο}_{i,l}$ and ${ο}_{i,k}$ indicate two possible state values that may occur in ***Q_i_*** respectively, *P*(*o_i,k_*|*o_i,l_*) indicates the transition probability from *o_i,l_* to *o_i,k_*, *N*(*o_i,l_*, *o_i,k_*) indicates the count of the event that the state transits from *o_i,k_* to *o_i,l_* and $\;\Psi_{i}=\left\{o_{i,1},o_{i,2},\cdot \cdot \cdot ,o_{i,\left|\Psi_{i}\right|}\right\}$ indicates the set of all possible state values, *i.e.*, $\forall q_{i,j}\in \Psi_{i}$. After calculated the transition probabilities between all states of the constructed PFSA, we can obtain the state transition probability matrix $\;\Pi_{i}$ defined by Equation (6):

(6) $$\Pi_{i}=\left[\begin{array}{ccc} p({ο}_{i,1}|{ο}_{i,1}) & \cdots & p({ο}_{i,1}|{ο}_{i,\left|\Psi_{i}\right|})\\ \vdots & \ddots & \vdots \\ p({ο}_{i,\left|\Psi_{i}\right|}|{ο}_{i,1}) & \cdots & p({ο}_{i,\left|\Psi_{i}\right|}|{ο}_{i,\left|\Psi_{i}\right|}) \end{array}\right]$$

After obtained the transition probability matrix, we calculate its left eigenvectors and generate one feature descriptor of the original PIR signals according to Equation (7):

(7) $$f_{i}=\sum_{j=1}^{\left|\Psi_{i}\right|}\nu_{j}$$

where *f_i_* indicates the feature descriptor generated from the *i*th symbol sequence and *v_j_* indicates the *j*th normalized eigenvector of $\;\Pi_{i}$. Through repeating this feature descriptor generation procedure on the five symbol sequences respectively, we can generate five feature descriptors for each PIR signal.

Because the IMF components are numerically orthogonal to each other [14] and the symbolization procedures of these IMF component are mutually independent (Section 3.1), the symbol sequences generated by the EMD-based symbolization method are mutually uncorrelated, and so do the feature descriptors.

### 3.3. Weighted Voting Classification Strategy

After generated five feature descriptors for the original PIR signal, next we need to fuse the feature information contained in the feature descriptors for classifying the original PIR signal. Therefore, inspired by the boosting algorithm [22], we propose a weighted voting classification strategy with five classifiers.

**Figure 8:**
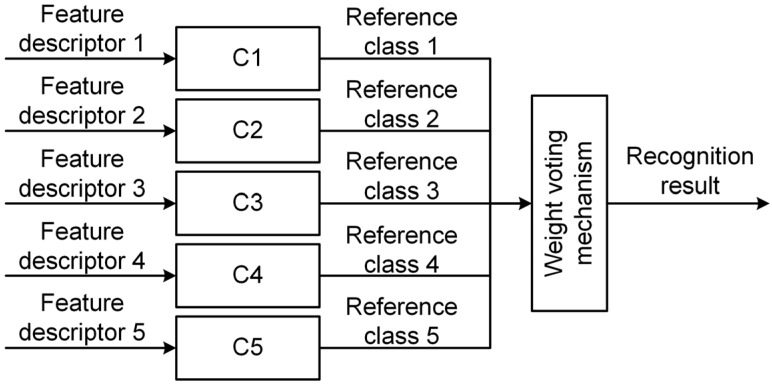
Construction of the weighted voting classification strategy, where *C_i_* indicates the *i*th classifier.

The proposed classification strategy is shown in Figure 8. First, we separately classify the generated feature descriptors with five classifiers and obtain five reference classes of the original PIR signal. Then, according to the generated reference classes, the five classifiers vote for the final class of the original PIR signal through a weighted voting mechanism.

Specifically, we assign higher voting weights to the classifiers with low classification errors, whereas the classifiers with high classification errors can only obtain a lower voting weights. In this way, a more accurate classifier can play a greater role in determining the final class of the original PIR signal. Algorithm 1 gives the voting weights determination procedure of the proposed classification strategy.

**Algorithm 1.** Voting weights determination procedure:   **Input:** A dataset of PIR signals **T** = {*x*_1_,*y*_1_),(*x*_2_,*y*_2_),…,(*x_N_*,*y_N_*)} with size of *N*, where *x_i_*∈**R***^n^* is the *i*th PIR signal and *y_i_*∈{–1,1} is the class of *x_i_*, −1 indicates “*nonhuman*” and 1 indicates “*human*”;   **Output:** A linear combination of five classifiers.   **Step 1.** Implement the proposed feature extraction method on dataset **T** to generate five feature sets: **F**_1_, **F**_2_,…,**F**_5_, where **F***_j_*={*f*_1*,j*_, *f*_2,*j*_,…,*f_N,j_*} and *f_i,j_* is the *j*th feature descriptor of the *i*th PIR signal in the dataset;   **Step 2.** From *j* = 1 to 5 do:
(a)Train the *j*th classifier and calculate its classification error *e_j_* defined by Equation (8) with the feature set **F***_j_* using the five-fold cross validation procedure recommended by [23], where *C_j_* indicates the discriminant function of the *j*th classifier and *I* is the indicator function:(8)$e_{j}=\frac{1}{N}\sum_{i=1}^{N}I\left(C_{j}\left(f_{i,j}\right)\neq y_{i}\right)$(b)Calculate the voting weight of the jth classifier using Equation (9) (9)$w_{j}=\frac{1}{2}\log\left(\left(1-e_{j}\right)/e_{j}\right)$   **Step 3.** Construct a liner combination with *C_1_*, *C_2_*, …, *C_5_*, and the final class of a PIR signal *x* can be determined according to Equation (10), where *FC* is the final class of *x*, *f_j_* is the *j*th feature descriptor of *x*, and *sign* indicates the signum function:(10)$FC=sign\left(\sum_{j=1}^{5}w_{j}C_{j}\left(f_{j}\right)\right)$

## 4. Experiments and Discussion

To verify the effectiveness of the proposed method, we establish two databases of human and nonhuman PIR signals. The contents of these two databases are shown in Table 1. Note that the PIR signals in Database 1 are collected from adults, dogs, and the warm wind generated by an air condition in different indoor environments, whereas those in Dataset 2 are collected from adults, dogs, and geese in different outdoor environments. All PIR signals are collected with a commercial PIR detector and a data acquisition card using a sampling frequency of 1000 Hz and a duration of 2 s. With these two databases, we perform multiple experiments to compare the performance of the proposed method, WPE [6], AR [7] and SDF-based [24] (without the wavelet preprocessing) feature extraction methods.

**Table 1:** Construction of human and nonhuman PIR signal database.

Database 1			Database 2		
Adults	Dogs	Warm wind	Adults	Dogs	Geese
200	200	200	200	200	200

### 4.1. Experiments on Database 1

The experiments implemented on Database 1 are used to verify the effectiveness of the proposed method for indoor intrusion detection. The reason why we choose the dog and warm wind as the nonhuman targets is that the false alarms of the PIR detector in the indoor environment are mainly caused by pets and heating apparatus.

**Figure 9:**
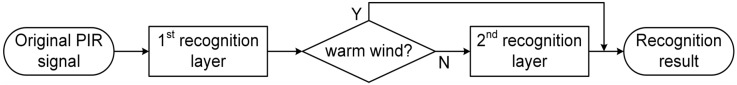
Two-layer recognition procedure for indoor intrusion detection.

Generally, it may be more reasonable to use a one-class classifier to implement the human and nonhuman recognition, but there is only a small range of disturbance sources of the PIR detector in the indoor environment, therefore, besides the one-class classifiers, we can also adopt the support vector machine (SVM) as the classifier of the proposed classification strategy for obtaining a higher classification accuracy. Because SVM is a binary classifier, as shown in Figure 9, we adopt a two-layer recognition procedure to deal with the three-class (*i.e.*, adult, dog and warm wind) classification problem. Each layer contains a feature extraction and classification procedure elaborated in Section 3. Because the characteristics of the warm wind’s PIR signals are quite different from the ones of adults and dogs, we use the first recognition layer to determine whether the input PIR signal is collected from warm wind, if not, we will use the second recognition layer to determine the specific class of the original PIR signal (*i.e.*, adult or dog).

#### 4.1.1. Voting Weights Determination

As described in Section 3.3, in the proposed classification strategy, the voting weights of the classifiers are directly calculated according to the voting weights determination procedure described in Section 3.3 rather than the wildly used grid searching algorithm. Therefore, to determine the voting weights of the classifiers, we first equally divide Database 1 into two datasets and term them as Subset 1 and Subset 2 respectively, then we use Subset 1 to execute the voting weights determination procedure and use Subset 2 to perform the comparative experiments.

To determine the voting weights of the classifiers in the first recognition layer, we categorize the adults’ and the dogs’ PIR signals in Subset 1 to a same class and label them as the positive samples, whereas the warm wind’s PIR signals are marked as the negative samples, and then we perform the Algorithm 1 described in Section 3.3 with all PIR signals in Subset 1. While in the voting weights determination procedure for the second recognition layer, we discard the warm wind’s PIR signals and just use the adult’s and dog’s PIR signals to perform the Algorithm 1. The classification errors of the classifiers in each recognition layer are summarized in Table 2, where *SVM_j_* indicates *j*th SVM of the weighted voting classification strategy and *RL_m_* indicates the *m*th recognition layer. According to the classification errors, we calculate the voting weights of the classifiers according to Equation (9), the voting weights are summarized in Table 3 and their sum has been normalized to one.

**Table 2:** Classification errors of the classifiers in each recognition layer for the recognition of adults, dogs and warm wind in the indoor environment.

*Classifier*	*SVM_1_*	*SVM_2_*	*SVM_3_*	*SVM_4_*	*SVM_5_*
*RL_1_*	1.50%	1.50%	6.50%	12.00%	7.00%
*RL_2_*	4.00%	2.50%	10.00%	17.50%	13.50%

**Table 3:** Voting weights of the classifiers in each recognition layer for the recognition of adults, dogs and warm wind in the indoor environment.

*Classifier*	*SVM_1_*	*SVM_2_*	*SVM_3_*	*SVM_4_*	*SVM_5_*
*RL_1_*	0.27	0.27	0.17	0.13	0.16
*RL_2_*	0.26	0.29	0.18	0.12	0.15

#### 4.1.2. Experimental Results and Discussion

After determined the voting weights, we use the leave-one-out cross validation procedure recommended by [23] to optimize another parameters (*i.e.*, the size of the alphabets) of the proposed method and testify its effectiveness with Subset 2. Besides the proposed method, we also implement the WPE, AR and SDF-based feature extraction method on Subset 2 for comparison. The parameter tuning and performance evaluation of these methods are also using the above-mentioned leave-one-out procedure. The experimental results are listed in Table 4 using the confusion matrix, where the rows are the actual classes and the columns are the predicted classes. The “Recall”, “Precision” and the “Recognition accuracy” of each method are summarized in Table 5.

We can see from Table 5 that, for the recognition of the warm wind, the proposed method only shows a slightly better performance than another three methods, but for the recognition of the adults and dogs, the proposed method shows a remarkably better performance that only two human targets are missed and no false alarm emerges.

**Table 4:** Recognition results of each method in the experiments executed on Database 1.

Methods	*Class*	Warm Wind	Dog	Human
	Warm wind	97	3	0
WPE	Dog	5	93	2
	Human	2	7	91
	Warm wind	99	1	0
AR	Dog	0	98	2
	Human	0	4	96
	Warm wind	93	7	0
SDF	Dog	4	87	9
	Human	0	15	85
**Our method**	**Warm wind**	**100**	**0**	**0**
	**Dog**	**0**	**100**	**0**
	**Human**	**0**	**2**	**98**

**Table 5:** Recall, precision and the overall recognition accuracy of each method in the comparative experiments on Database 1.

Recognition Method	Warm Wind		Dogs		Adults		Recognition Accuracy
	Recall	Precision	Recall	Precision	Recall	Precision	
WPE	97.00%	93.27%	93.00%	90.29%	91.00%	97.85%	93.67%
AR	99.00%	100%	98.00%	95.15%	96.00%	97.96%	97.67%
SDF	93.00%	95.88%	87.00%	79.82%	85.00%	90.43%	88.33%
**Our method**	**100.00%**	**100.00%**	**100.00%**	**98.04%**	**98.00%**	**100.00%**	**99.33%**

EMD is a self-adaptive signal decomposition method, so unlike the wavelet decomposition, it has no need of any base functions [14], so the proposed method can avoid the performance degradation caused by a bad choice of wavelet bases. EMD is also a suitable tool for non-stationary signal processing, therefore, the proposed method can provide a more reliable recognition result than AR-based feature extraction because sometimes the PIR signals are non-stationary. As for SDF, it has the advantage of low computational complexity and memory requirement [9], but because of the coarse graining of the original PIR signal, it may loss the feature information contained in the signal details, which are closely related to the body shape features of the human and nonhuman targets. The proposed method can be viewed as an improvement of SDF, because EMD makes it possible to specially analysis the detailed components of the original PIR signal, the proposed method can effectively extract the discriminable features of human and nonhuman PIR signals. However, we cannot claim that our method is better than another three methods for any applications, because the introduction of EMD also cause an increment of computational complexity and memory requirement. There should be a comprehensive consideration between the requirement of recognition accuracy and the computing speed.

### 4.2. Experiment on Database 2

The experiments implemented on Database 2 are to verify the effectiveness of the proposed method for outdoor pedestrian detection. In our application, the most likely occurred nonhuman targets are dogs and geese, or another wild animals with similar body sizes and shapes, that’s the reason why we choose doge and geese as the nonhuman subjects in our experiments.

**Figure 10:**
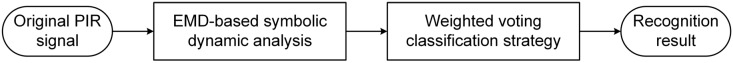
Recognition procedure of the outdoor pedestrian detection.

Because dog’s and geese’s PIR signals are quite similar with each other, but they are relatively distinctive from human PIR ones, we can combine the dog’s and geese’s PIR signals together as an outlier class and classify them from the human PIR signals using a one-layer recognition procedure shown in Figure 10.

#### 4.2.1. Voting Weights Determination

Similar to the experiments on Database 1, to determinate the voting weights of the classifiers in the weighted voting classification strategy, we equally divide the Database 2 into two subsets and term them as Subset 3 and Subset 4, respectively. Subset 3 will be used to perform the voting weights determination procedure elaborated in Section 3.3, and Subset 4 will be used to verify the effectiveness of the proposed method.

To perform the voting weights determination procedure, we combine the dog and goose’s PIR signals in Subset 3 together and label them as the negative samples. Then we execute the Algorithm 1 with all PIR signals in Subset 3. The classification errors of the classifiers are summarized in Table 6, where *SVM_j_* indicates *j*th SVM of the weighted voting classification strategy and *RL_m_* indicates the *m*th recognition layer. According to these classification errors, we calculate the voting weights of the classifiers using Equation (9), the normalized voting weights are listed in Table 7.

**Table 6:** Classification errors of the classifiers for the recognition of adults, dogs and geese in the outdoor environment.

Classifier	*SVM_1_*	*SVM_2_*	*SVM_3_*	*SVM_4_*	*SVM_5_*
Error	2.00%	3.00%	10.50%	17.50%	17.50%

**Table 7:** Voting weights of the classifiers for the recognition of adults, dogs and geese in the outdoor environment.

Classifier	SVM_1_	SVM_2_	SVM_3_	SVM_4_	SVM_5_
Weight	0.31	0.28	0.17	0.12	0.12

#### 4.2.2. Experimental Results and Discussion

After determined the voting weights, to verify the effectiveness of the proposed method for outdoor pedestrian detection, we implement the proposed method on Subset 4 and compare its performance with the WPE, AR-based and SDF-based feature extraction method, the parameter selection and performance evaluation of these methods are identical with that in Section 4.1. The classification results are summarized in Table 8. The recall, precision and the overall recognition accuracy of each method are listed in Table 9.

**Table 8:** Recognition results of each method in the experiments executed on Database 2.

Methods	*Class*	Human	Nonhuman
WPE	Human	90	10
	Nonhuman	6	194
AR	Human	95	5
	Nonhuman	4	196
SDF	Human	84	16
	Nonhuman	8	192
**Our method**	**Human**	**97**	**3**
	**Nonhuman**	**0**	**200**

**Table 9:** Recall, precision and the overall recognition accuracy of each method in the comparative experiments on Database 2.

Recognition Method	Human		Nonhuman		Recognition Accuracy
	Recall	Precision	Recall	Precision	
WPE	90.00%	93.75%	97.00%	95.10%	94.67%
AR	95.00%	95.96%	98.00%	97.51%	97.00%
SDF	84.00%	91.30%	96.00%	92.31%	92.00%
**Our method**	**97.00%**	**100.00%**	**100.00%**	**98.52%**	**99.00%**

We can see from Table 8 and Table 9 that the proposed method obtains the highest recognition accuracy that only three human targets are missed and no false alarm emerges.

Although the most common nonhuman subjects in our applications are dogs and geese, or another wild animals with similar body shapes, there may be more disturbance sources in other applications. Therefore, we recommend that readers use a flexible strategy to deal with the multiclass classification problem, for example, combining all nonhuman targets together and classifying them from the human ones, or just adding more layers into the recognition procedure elaborated in Section 4.1. Besides, the one-class classifiers, such as support vector data description (SVDD) [25], are also recommended.

## 5. Conclusions

For reducing PIR detector false alarms, after analyzing the inherent differences between human and nonhuman PIR signals using a mathematical model of a PIR detector, we propose an EMD-based symbolization method for generating five symbol sequences with the detailed feature information of the original PIR signals. Then, we construct the multiple PFSA based on the generated symbol sequences for extracting five feature descriptors of the original PIR signal. Third, we used a weighted voting classification strategy to fuse the features represented by the feature descriptors and then classify the original PIR signals. To verify the effectiveness of the proposed method, we executed comparative experiments on two databases with the proposed method, WPE, SDF and AR-based feature extraction methods. The experimental results show that the proposed method can effectively reduce the false alarms of a PIR detector.
